# Biomarkers for Pediatric Pulmonary Arterial Hypertension – A Call to Collaborate

**DOI:** 10.3389/fped.2014.00007

**Published:** 2014-02-03

**Authors:** Kelley L. Colvin, Melanie J. Dufva, Ryan P. Delaney, D. Dunbar Ivy, Kurt R. Stenmark, Michael E. Yeager

**Affiliations:** ^1^Department of Bioengineering, University of Colorado Denver, Aurora, CO, USA; ^2^Department of Pediatrics-Critical Care, University of Colorado Denver, Aurora, CO, USA; ^3^Cardiovascular Pulmonary Research, University of Colorado Denver, Aurora, CO, USA; ^4^Linda Crnic Institute for Down Syndrome, University of Colorado Denver, Aurora, CO, USA; ^5^Children’s Hospital Denver, Aurora, CO, USA

**Keywords:** pulmonary arterial hypertension, pediatric, biomarkers, imaging, magnetic resonance, echocardiography, right ventricle

## Abstract

Therapeutic approaches in pediatric pulmonary arterial hypertension (PAH) are based primarily on clinician experience, in contrast to the evidence-based approach in adults with pulmonary hypertension. There is a clear and present need for non-invasive and objective biomarkers to guide the accurate diagnosis, treatment, and prognosis of this disease in children. The multifaceted spectrum of disease, clinical presentation, and association with other diseases makes this a formidable challenge. However, as more progress is being made in the understanding and management of adult PAH, the potential to apply this knowledge to children has never been greater. This review explores the state of the art with regard to non-invasive biomarkers in PAH, with an eye toward those adult PAH biomarkers potentially suitable for application in pediatric PAH.

## Introduction

Our understanding of pulmonary hypertension (PH) in children has been hampered over the years by a number of factors. Despite the important benefits of off-label application to children of PH therapies originally developed for adults, the disease remains lethal. The study of PH in children is further complicated by additional factors such as the complexity of accurate diagnosis, the multifactorial nature of the disease, and a relatively poor understanding of the natural history of the disease. The need for earlier detection, more accurate and sensitive biomarkers of disease and disease progression, and a personalized approach to therapy cannot be overstated. To date, biomarker reviews for PH have focused on adults. This review focuses on the unrealized promise of biomarkers in pediatric PH, their potential to improve our ability to treat PH, and the capacity for adult biomarkers of PH to be applied to children.

## Definition and Classification of Pediatric Pulmonary Hypertension

Pulmonary hypertension is a condition characterized by increased blood pressure and resistance in the arterial vasculature of the lung as the result of numerous pathological mechanisms, eventually culminating in right ventricular failure. The development of the disease may be linked to other diseases as a derived secondary disease, or may develop solitarily with known etiology, or may be idiopathic (1). PH may develop in both children and adults, regardless of age. Currently, as elaborated by the World Health Organization Dana Point 2008 Clinical Classification system, there are five categories of PH, with the most common form of childhood PH (amounting to ~90% of cases) occurring in Group I (1). Group I, denoted as pulmonary arterial hypertension (PAH), includes idiopathic (IPAH), familial (FPAH), PAH associated with venous and/or capillary disorders, or associated with other diseases (APAH), including HIV infection, drugs, toxins, congenital shunts between pulmonary and systemic circulation, collagen vascular disease, and others. Groups II–V are categorized as PH associated left heart disease, PH associated with lung diseases/hypoxia, PH due to embolic and/or chronic thrombotic disease, and PH associated with miscellaneous conditions, respectively.

Current standards define PAH in adults and children in a similar way, with specified parameters of a mean pulmonary artery pressure ≥25 mmHg, a normal capillary wedge pressure ≤15 mmHg, and an increased pulmonary vascular resistance (PVR) (2). The minimum value for increased PVR remains controversial, especially in pediatric patients, where PAH caused by left-to-right congenital shunts is common, and in such cases no significant increase in PVR is observed (3). Therefore, most experts suggest a PVRI ≥3 WUm^2^ for diagnosis of pediatric PAH (3). When left untreated, children with PAH are predicted to have lower survival rates and poorer prognosis than adults (4). Abnormal lung and cardiac development is known to play a factor in pediatric PAH, in which left-to-right shunts due to congenital heart defects causes increased flow in the pulmonary vasculature, known as the Eisenmenger syndrome (5). Studies have shown the pathophysiology for PAH to be comparable between children and adults (6–8), yet there remains limited data on the efficacy of current adult therapies extrapolated to children. Pediatric PAH is a rare, multifactorial disease, with no current cure. Therefore, there is a need to standardize prognostic procedures for the establishment of early and appropriate therapeutic responses.

Clinically, children with PAH show various symptoms, may be non-specific, and are dependent upon age. Symptoms include shortness of breath, exercise fatigue, respiratory symptoms and chest pain (9). Current diagnostic methods include echocardiography (Echo), exercise testing, right heart catheterization (RHC), and vasodilator testing for both adults and children (10). In children in whom RHC is deemed unsuitable, the diagnosis is usually reached as a validated consensus from a team of pediatric PH specialists. And subsequent therapy is based on similar algorithms used in adults as well as the experience of the clinical team (11).

## Epidemiology and Worldwide Health Burden of PH

For adults with PAH, studies have reported an incidence of 7.6 per million cases of adults per year and a prevalence of 52 cases per million (12). Limited data are available on disease registries of pediatric PAH, due to small patient numbers, and because earlier registries lack standardized assessments (4). However, recently, a number of studies have been completed for disease registry and to further improve understanding of the disease epidemiology in children (13). PAH in children is rarer than in adults. Data from a registry in the Netherlands reported the incidence of pediatric PAH to be 63.7 cases per million children, with a majority of those cases being PAH diagnosed (57.9 cases per million children) (14), a comparable incidence to that seen in adults. In a study conducted by the UK Pulmonary Hypertension Service for Children, survival rates for PAH diagnosed children, treated with modern therapies (mono- or combination therapies, or disease-specific therapy, including treatment with calcium channel blockers, bosentan, and intravenous epoprostenol), were reported at ~90.5, 82.8, and 64.2%, at 1, 3, and 5 years, respectively (15). Another study conducted by the Registry to Evaluate Early and Long-Term PAH Disease Management (REVEAL) reported survival rates of IPAH/FPAH and APAH-CHD children, treated with etiologic specific therapies, at 96, 84, and 74% for 1, 3, and 5 years, respectively (16). These data are supported by results in similar studies (14, 17, 18). Data show varying survival estimate differences between IPAH and APAH patients (14–16), which may be due to limited sample sizes and various subgrouping analysis. Compared to adults, data from the Tracking Outcomes of Pediatric Pulmonary Hypertension (TOPP) showed a higher distribution of APAH associated with CHD in children, with a very low occurrence of PAH associated with tissue diseases, HIV, drugs, and portopulmonary hypertension (19). A higher occurrence of the disease in children has been observed in females than males at a 2:1 ratio (20), which is lower compared to the 4:1 ratio seen in adult patients (21).

The recent data from these registries report preliminary findings on the efficacy of adult therapies in children, with positive improvements in the prognosis. Though adults and children with PAH have shown similar pathobiology and clinical responses to treatment therapy, much is still unknown about pediatric PAH, and full elucidation of the differences between adults and children still remains.

## Biomarker Definition

The National Institutes of Health defines a biomarker as: “a characteristic that is objectively measured and evaluated as an indicator of normal biologic processes, pathogenic processes, or pharmacologic responses to therapeutic intervention” (22). This broad definition is particularly apt for pediatric diseases, which may involve developmental disturbances with and without pathogenic and pharmacologic processes. Recently, several excellent reviews on biomarkers in adult PAH have been published (23–25). This review focuses on those biomarkers that have been proposed for, or may have specific relevance to, pediatric PAH.

## General Biochemical Classes of Pulmonary Hypertension Biomarkers

**Figure 1:**
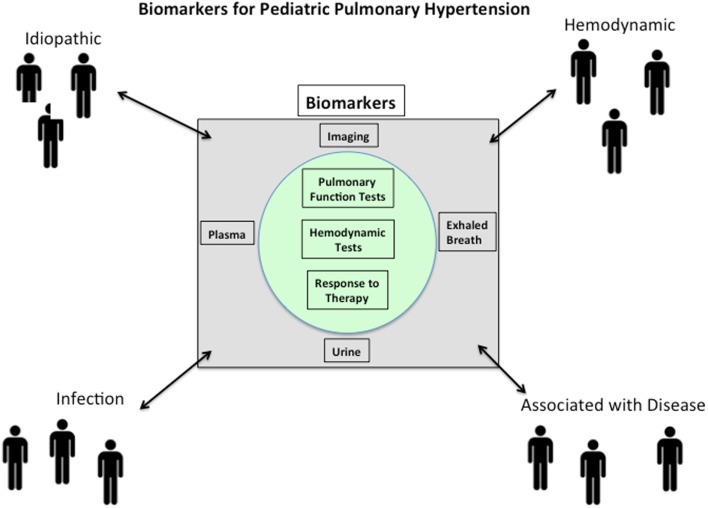
**Pulmonary arterial hypertension (PAH) is a syndrome that is idiopathic in origin or arises in association with a large number of disease processes**. Biomarkers for PAH should correlate in some fashion to a hemodynamic measurement such as PA pressure, PA wedge pressure, pulmonary vascular resistance, etc., and/or to a clinical measurement such as survival, pulmonary function test, etc. Since the pathobiology of PAH is multifactorial and complex, it should not be surprising that potential biomarkers will likely reflect aspects of the underlying disease process.

The ultimate biomarker for any disease would be one that involves a procedure that is easy to perform, painless, inexpensive, while offering high specificity, and low rates of false negatives and positives. For a biomarker to be effective in a pediatric population, additional factors such as patient compliance must be considered. During the typical patient workup, many routine tests are ordered that can serve as biomarkers. Some sources, such as plasma, could potentially be screened to simultaneously measure panels of biomarkers. Indeed, there is unlikely to be any single biomarker for PAH that perfectly satisfies all of these requirements. Furthermore, in the age of “personalized medicine,” biomarkers may be of better value when measured and compared longitudinally in a single patient, rather than compared to others as absolute values at single time points. As a starting point, PAH is a hemodynamic definition, representing a spectrum of diseases that ultimately leads to right heart failure. Therefore, biomarkers for PAH should correlate in some fashion to a hemodynamic measurement such as PA pressure, PA wedge pressure, PVR, etc., and/or to a clinical measurement such as survival, pulmonary function test, etc. (Figure 1) (23).

The pathobiology of PAH is multifactorial and complex. Therefore, it should not be surprising that potential biomarkers likely should reflect aspects of the underlying disease process (Figure 1). Damage to pulmonary endothelium and ongoing endothelial cell (EC) dysfunction is thought to play a fundamental role in the pathogenesis of PAH (26). Many of the therapies used in patients with PH relate to the biology of dysfunctional endothelium by targeting prostaglandins, nitric oxide, and endothelins (23). Inflammation in the lungs and right ventricle is thought to play significant roles in the pathobiology of PAH (27, 28). Mast cells, monocytes, macrophages, T cells, and B cells have all been shown to participate at some level in PAH (29). Both innate and acquired immune cells are rich sources of cytokines and chemokines that have been reported to be prognostic biomarker in adult PAH (23). The larger question of whether these inflammatory markers are indicators of general inflammation and oxidative stress or whether they are specific for pulmonary vascular disease and/or right ventricle dysfunction is not known. In addition, significant variability in the levels of some cytokines (ex: IL-6) has been reported (30, 31), likely due to differences in patient cohorts and in assay methodology. These reproducibility issues must be resolved before their widespread acceptance and use in the clinic. Finally, biomarkers of heart dysfunction have been increasingly used to evaluate and often prognosticate PAH. This is evidenced by a PubMed search conducted in January 2014 using the terms “biomarkers right ventricle pulmonary hypertension,” which resulted in 90 hits with 52/90 published in the last 5 years [four excellent examples (32–35)]. Measurement of atria natriuretic peptide (ANP) and most especially B-type natriuretic peptide (BNP) and N-terminal (NT)-proBNP provide important and reproducible prognostic markers with regard to cardiac function vis-à-vis blood volume and pressure (36).

## Specific Classes of Pulmonary Hypertension Biomarkers

For the purposes of this review, we exclude invasive hemodynamics but include as a biomarker, any modality that yields data that can aid in assessment of PAH at any point in its course. Such a perspective admittedly ignores real-world limitations of resources, expertise, and the recruitment of sufficiently large enough patient cohorts. Specific classes of biomarkers for PAH can be organized into those arising from imaging tests, circulating biomarkers, exhaled breath, and urinary biomarkers.

### Imaging tests

#### Echocardiography

The gold standard for assessment of PAH is RHC, in which mean pulmonary artery pressure (pa), cardiac output, and PVR measurements can be obtained (37). Three recent reviews provide excellent summaries of RHC (38–40). Prior to invasive hemodynamics, an echocardiogram is almost always performed. Echo is an important screening tool for the diagnosis of PAH, and is also the standard of care for non-invasive monitoring of PH progression. Echocardiographic findings, although subject to significant operator variability (41), reliably provide several estimates of hemodynamic function that closely correlate with measurements obtained by RHC. A large variety of estimates of RV function can be made, depending on assumptions of RV geometry. Flattening and inversion of the inter-ventricular septum toward the left ventricle is highly suggestive of PAH (42). Detailed echocardiographic methodology and the equations used for calculation of variables are detailed elsewhere (43). Recently, the importance of Doppler Echo based measurement of the acceleration time of pulmonary flow has become an important metric, as has the tricuspid annular plane systolic excursion (TAPSE) (44). Decreased TAPSE has been associated with poor prognosis in patients with dilated cardiomyopathy, and may have correlation with pa and PVR (45). Recently, tissue Doppler imaging (TDI) has been used to predict adverse outcome in children with IPAH (46). This approach measures three waveforms that represent the cardiac cycle: systolic myocardial velocity (Sm), early diastolic myocardial relaxation velocity (Em), and late diastolic myocardial velocity associated with atrial contraction (Am). This prospective study compared 51 children with IPAH to 51 controls and found that tricuspid Em had higher correlation with plasma BNP levels and hemodynamics than tricuspid Sm. These findings differed with those from adults (47–49), but, importantly, support our thesis that biomarkers in adults with PH should be systematically and comparatively examined in children with PAH. As with nearly all studies of pediatric PAH, interpretation of the study is constrained by the relatively small sample size. In addition, the patients were placed on vasodilators suited to their particular clinical portraits and all survived, and so the ability of TDI to predict mortality was not assessed. Nevertheless, this prospective study illustrates the power of non-invasive testing by Echo as part of the biomarker toolkit of the pediatric cardiologist.

#### Computed tomography

Multi-detector computed tomography (CT) is used routinely to assess both cardiovascular and lung parenchymal changes in patients with PAH. With the advent of respiration-timed CT gating with electrocardiography, the technique has vastly improved (50). As much as its value has improved, correlation with measurements such as main pulmonary artery diameter to pa is variable (51–53). However, several studies have used CT imaging to establish the differential diagnosis of PAH by ruling in or out associated pathologies such as pulmonary thromboembolism or pulmonary fibrosis. Recent work has begun to demonstrate good correlation between functional parameters such as right pulmonary artery wall distensibility and diagnosis of PAH (54). As respiration gating improves along with increasing image detail, CT will continue to be an important assessment tool for the diagnosis of PH. By far the largest drawback to the use of CT is exposure to X rays. Not only does this limit the capacity for CT to provide longitudinal study data, its use in children is problematic and the benefits must be weighed against exposure time. For example, the contrast agents used are associated with kidney injury (55). Furthermore, patient compliance (inability to hold still, claustrophobia, etc.) in a pediatric population can be problematic.

#### Single photon emission computed tomography

Single photon emission computed tomography (SPECT) is a variation in CT in which gamma rays emitted from an injected radionuclide source are detected by a rotating gamma camera (56). The technique allows for three-dimensional reconstruction, and when used with specific radioligands, tissues of interest can be closely scrutinized. Of particular interest is the ability to measure true perfusion with Technetium-99m labeled macro-aggregated albumin (57). Unfortunately, acquisition of SPECT images typically takes several minutes and breathing during acquisition affects image resolution, yet has been performed successfully in animal models of PAH (58). As with CT, respiration gating improves image quality. In any case, the ability to perform SPECT in pediatric lung disease is limited, but the promise of three-dimensional reconstructions of perfused lung vasculature is enticing.

#### Positron emission tomography

Positron emission tomography (PET) has seen a dramatic increase in use over the past decade in the setting of PH. Recently, it was shown that PAH hearts have pathologic glycolytic metabolism that is quantitatively related to cardiac dysfunction over time (59). Interestingly, the FDG uptake observed also seemed to correlate with circulating CD34+CD133+ cells (a class of biomarker discussed in the next section). Breath-gated PET had moderate-to-high correlation with cardiac magnetic resonance (CMR) imaging and CT in the assessments of RV volume and ejection fraction (60). FDG uptake by the RV reflects the severity of PVR in PAH. Increased RV FDG uptake is a marker of poor prognosis in IPAH and is reduced in patients receiving effective therapy (61). What is somewhat unclear is the extent to which FDG PET in patients with PAH truly measures increased cellular metabolism or reflects inflammatory processes. One study recently reported that there were no correlations between (18) FDG uptake and hsCRP or inflammatory cytokine levels in PAH patients. However, NT-ProBNP correlated with RV uptake in those with PH suggesting that FDG PET may be a good biomarker for RV dysfunction (62). In experimental PH, Glut1 up-regulation in proliferating vascular cells in PAH accounts for increased lung FDG PET uptake. FDG PET is sensitive to mild PAH and can monitor therapeutic changes in the vasculature (63). Collectively, these studies strongly suggest that metabolic imaging may be useful in therapeutic monitoring of PAH patients. In adults, PET appears to be a suitable method for assessing RV function and myocardial glucose metabolism in patients with PAH, as well as lung metabolic and cellular proliferation.

For CT, SPECT, and PET, important considerations regarding radiation dose vis-à-vis diagnostic benefits must be carefully weighed. However, the value of nuclear medicine in pediatrics is well established, as has standard dosimetry for a large variety of radiopharmaceuticals (64).

#### Magnetic resonance imaging

Cardiac magnetic resonance imaging (MRI) has rapidly evolved to be a powerful tool to measure right ventricular morphology, and volumes and ejection fractions (65–69). The ventricular mass index, which is the quotient of right ventricular mass over left ventricular mass, is highly sensitive and specific for prognosticating PAH [reviewed in Ref. (70)]. Furthermore, its advantages in spatial resolution, lower intra-operator variability, and true three-dimensionality make it the gold standard for RV study over Echo (71). Cardiac MRI has been successfully used to determine RV stroke volume index, RV ejection fraction, RV mass, RV isovolumic relaxation time, leftward ventricular septal bowing, and left ventricular ejection fraction (72). With regard to children with PH, MR can be performed safely and effectively. In one study of 26 subjects, markedly abnormal RVs were found (73). One year later, no change in cardiac MR parameter was observed, which highlights the advantage of MR for serial testing. Additional morphological and functional parameters could be measured in children to include inter-ventricular septum configuration, stress testing, fibrosis (with late gadolinium enhancement), and pulmonary circulation testing (74). A detailed treatment of the wide range of potential uses for MRI in normal and PAH lung has been published (75). Recently, the prognostic value of MR was evaluated in 100 children with PAH, of which 60 were diagnosed with IPAH (76). Almost all parameters assessed by MR correlated with clinical metrics of disease severity and had strong correlation to invasively measured pa. Detailed MRI assessment of heart and lung function in children with PH at initial workup and serially in the course of treatment will be an increasingly important tool for the clinician.

### Circulating biomarkers

Blood is a potentially rich source of biomarkers in the setting of pediatric PAH. Since PAH is a multifactorial and complex vascular disorder, it stands to reason that a variety of biomarkers would be present arising from sources related to ongoing processes of inflammation, coagulation, and ventricular strain. For this review, we have divided blood biomarkers into compartments of cells and non-cells (plasma or serum). Within the cellular compartment, leukocytes, erythrocytes, and platelets are routinely analyzed in the hospital laboratory setting. Recent studies have measured microparticles, circulating endothelial cells (CECs), and endothelial progenitor cells (EPCs) in peripheral and/or central blood. In addition, a substantial number of chemokines, cytokines, and even RNAs have been measured in the blood plasma and correlated to physiological and clinical parameters of PAH, mostly in adults.

#### Cells and microparticles

Many disease states are characterized by increase or decrease in circulating cells, often those involved in inflammation (77) or angiogenic responses to injury/repair (78). PH, at least in adults, is associated with EC dysfunction (79). CEC as well as EPC are readily identifiable in adults with PAH (80, 81). Microparticles, defined as plasma membrane fragments between 0.1 and 1 μm in diameter, are released during a number of physiologic and pathophysiologic conditions by numerous cell types (82). Recently, CECs were prospectively measured in children with IPAH and PAH secondary to congenital heart disease, before and after treatment (83). Importantly, rising CEC counts preceded clinical deterioration, suggesting that CECs may be an important tool to anticipate clinical worsening. Another study on endothelial biomarkers reported elevated levels of circulating ECs associated with reversible APAH-CHD in children, suggesting that endothelial dysfunction and damage play an important role in angiogenesis (84). This study also included analysis of soluble markers, and results showed that levels of angiogenic cytokine, inflammation, or endothelial microparticles were not predictors of PAH reversibility. Additional studies by Smadja et al. showed that harvested endothelial colony forming cells (ECFCs) from peripheral blood of children with IPAH and APAH-CHD had a hyperproliferative phenotype with enhanced angiogenic potential when treated with prostanoid therapy (treprostinil) in a nude preclinical mouse model of limb ischemia (85). These results provide support for the importance of EPCs in vascular repair in pediatric PAH. On the other hand, some studies have failed to establish any correlation between circulating EPC and pa (86). Small sample sizes, observer bias, or differences in technical approach may explain such disparate results. Flow cytometric assessment of circulating microparticles derived from platelet and erythrocytes in young thalassemia major patients positively correlated to markers related to PH and aortic wall stiffness (87). In a small study, microparticles form PAH patients were shown to have increased CD39 nucleotidase activity (88). Thus, circulating vascular cells, or microparticles derived from them, appear to be variable in number in PAH patients, may or may not correlate to clinical values, and may be involved in the pathogenesis. Whether measured in children or adult with PAH, a multi-center study with a large cohort, using a standardized methodology for a specified set of markers, should definitively establish the utility of vascular cell measurement as a biomarker.

In addition to vascular cells, inflammatory cells can be measured in peripheral blood. Deficiencies in natural killer cells and cytotoxic CD8+ T cells may portend risk of death in PAH patients (89). Compared to controls, individuals with PH and pulmonary veno-occlusive disease (PVOD) have decreased circulating T lymphocytes (90). In that study, epigenetic alterations of the granulysin gene discriminated PVOD from PAH. In yet another study, peripheral blood mononuclear cells (PBMCs) were purified and analyzed for expression of endoplasmic reticulum stress and unfolded protein response markers (91). Compared to controls, PAH patients’ PBMC had higher expression of these markers, which in the case of the unfolded protein response effectors DnaJB and BiP, correlated to inflammation (IL-6 levels) and disease severity (pa). Importantly, these studies seem to corroborate an emerging concept of cell stress in PAH that is being borne out in animal models of PAH (92). These results provide evidence that analysis of circulating white blood cells by flow cytometric quantification and subsequent gene expression characterization represent potentially important blood tests that could be integratively applied to current diagnostic and prognostic workups in children with PAH. Indeed, elevations of subsets of monocytic cell subsets have been identified in children with PAH (93, 94). Such studies, if confirmed in additional cohorts, could be prospectively expanded and performed serially to establish correlations to inflammation, disease progression, and response to therapy. During the clinical workup, numerous opportunities are available to measure circulating cells and microparticles, either centrally at catheterization or peripherally.

#### Plasma

The plasma has been investigated in both adult and pediatric PAH patients for a large number of biomolecules, including proteins, vitamins, and nucleic acids. As with circulating cells, it is thought that biomolecules in the plasma likely reflect underlying disease processes of lung vascular cell dysfunction (95), ventricular damage (96), and inflammation (97).

#### Plasma proteins

As mentioned above, NT-proBNP is probably the most studied (measured) plasma protein biomarker in PAH. In children, as is the case with adults, NT-proBNP has prognostic value (98, 99). The levels of NT-proBNP have been reported to be lower in children with PH than in adults (100, 101), a finding which seems to correspond with a decreased incidence of right heart failure in children vs. adult patients. Similarly, cardiac troponin T is an independent marker of mortality (102). A critically important aspect to consider in the pediatric PAH patient is age and/or stage of development. Indeed, children are not “small adults.” Developmental programs driving lung vessels are thought to be perturbed in PAH, and decreased plasma vascular endothelial growth factor (VEGF) in infants with PAH may be a reflection of such disturbances (103). Recent proteomic studies have demonstrated clear feasibility in measuring plasma interleukins (ILs), acute phase proteins, and growth factors (104, 105). Some of these plasma proteins may simply reflect systemic inflammation (serum amyloid A), but may also be mechanistically linked to control of inflammatory cell phenotype (serum amyloid P). For example, mast cells have long been implicated in the pathobiology of PH and efforts to understand their specific contributions have recently been re-energized (29). Growth differentiation factor (GDF)-15 (106), Endothelins (107), and C-reactive protein (108) appear promising as biomarkers in adult PH, at least with respect to inflammation. Recently, blood and urine leukotriene E-4, proangiogenic factors, mast cell numbers, and mast cell tryptase levels were higher in PAH patients, and receded subsequent to treatment with mast cell inhibitors cromolyn and fexofenadine (109). However, these declines were not linked to clinical improvement. Nevertheless, this study provides a representative framework to perform a comprehensive (multicompartment, multi-target) biomarker analysis that could be applied to children with PAH. Another study analyzing the relationship between hemodynamics and serum levels of intracellular adhesion molecule 1 (sICAM-1) showed that levels are elevated in children with APAH-CHD compared to children with only congenital heart disease, with mean pulmonary arterial pressure being the strongest independent predictor (110). Collectively, a large number of plasma proteins could be systematically evaluated in children with PAH. We suggest a multiplex platform, as we have used (104), but one in which the protein targets have been customized to a “PH plasma proteome signature” likely comprised of IL-6, GFF-15, sICAM-1, endothelins, serum amyloid A, and serum amyloid P. High accuracy/high precision multiplex assay platforms are already routinely capable of measuring >84 analytes with appropriate controls on a single 96-well plate. Because of this large analytic capacity, the choice of biomarkers to analyze might be less important as the choice to systematically adopt it. Development of close collaboration between biotechnology industry, academia, and biopharma seems particularly suited for the task of biomarker selection, analysis, and scope of interpretation. We offer a comprehensive recommendation for specific biomarkers in the conclusion section of this review.

#### Plasma non-proteins

There have been a large number of studies that have identified differences in levels of plasma non-proteins between PAH patients, PAH patient sub-groups, and controls [reviewed in Ref. (111)]. For this review, we have focused on vitamins and RNA species as non-protein biomarkers, but the complete list is much longer and beyond our scope here.

##### Vitamins

Vitamins are a large and diverse group of organic compounds that are essential for normal growth and nutrition and are required in small quantities in the diet because they cannot be synthesized by the body. There have been a large number of studies that have identified differences in plasma vitamin levels between PAH patients, PAH patient sub-groups, and controls. Curiously, there are very few reviews available that summarize potential roles of vitamins in PAH. For one example, Vitamin D levels affect the renin–angiotensin–aldosterone system, which, in turn, affects the cardiovascular system. Low Vitamin D levels correlated with higher systolic PA pressure (112). As a large number of PAH patients are therapeutically anti-coagulated, appropriate levels of Vitamin K are critically important due to major bleeding risks (113). Vitamin B12 (cobalamin C) deficiency may be associated with, or predispose individual to, pediatric PAH (114). Vitamin B1 (thiamine) deficiency is a cause of wet (cardiac) beriberi (115), which often manifests with symptoms of PAH (116). Finally, Vitamin C deficiency (117), elevated plasma copper (118), and iron deficiency (119) all appear to be associated with the development of PH. In the case of iron, its measurement as a biomarker can be extended by measurement of red cell distribution width, which is predictive for mortality in PAH patients (120). Unfortunately, the studies examining levels of vitamins in the setting of PAH are sporadic and involve small numbers and/or anecdotal cases. Furthermore, the efficacy of vitamin therapies (nutraceuticals) in PAH is questionable. With regard to children, overall nutrition is a critical component of healthy development, and can vary widely based on a number of factors, including socioeconomic issues. Here, we are not endorsing the therapeutic use of vitamins. We merely suggest that measurement of vitamins could represent powerful biomarker tools for the detection and management of PAH, particularly in kids. It could be argued that vitamins cannot serve as biomarkers because they simply reflect nutritional adherence. In the strictest sense, vitamin D is not truly an essential dietary vitamin since it can be synthesized from stored skin cholesterol in adequate amounts by most mammals exposed to sunlight. Similarly, vitamin A is stored as retinol or as retinyl ester, can be converted to retinoic acid, and a role of retinoids in PH has been investigated (121). Thus, vitamin deficiencies have been shown to be associated with PH, in some cases precede development of PH, and their levels in anatomical compartments do not directly reflect nutritional adherence.

##### RNA

MicroRNAs (miRNAs) are non-coding single stranded RNAs that vary in length between 19 and 25 nt. Their function is to “fine-tune” the regulation of gene expression by affecting mRNA stability and translation into protein (122). MicroRNAs are implicated in lung diseases and have been identified as attractive biomarkers due to their accessibility and stability in bodily fluids (123). Circulating miRNAs are thought to be released from injured cells, though this is not always the case (124). At the time of this writing, there have been no studies measuring blood plasma miRNAs in children with PH. However, several studies have identified altered levels of miRNAs in buffy coat cells from patients with PAH (125), some of which showed good correlation to the disease severity. Additional studies have identified other miRNAs and, in animal models, manipulation of mir-204 (126) and mir-21 (127) showed dramatic therapeutic potential. These studies point to the urgent need to measure miRNAs in plasma and buffy cot cells form children with PAH. As has been a common theme throughout this review, caution must be exercised in the interpretation of any biomarker measurements in children. In the case of miRNAs, differential levels may be related to stage of development, gender differences, etc. Nevertheless, miRNAs may prove to be important and novel biomarkers in pediatric PAH.

### Exhaled breath biomarkers

Due its non-invasive nature, breath analysis has the potential to be a simple and convenient alternative to traditional biomarkers sources such as blood. Over 40 years ago, Linus Pauling used gas chromatography to identify volatile organic compounds (VOCs) in exhaled breath (128). The use of breath biomarkers has been standardized by law enforcement to measure ethanol consumption in automobile drivers. Exhaled breath consists of ~500 mL of air, of which the first 150 mL does not participate in gas exchange (129). To detect VOC at the parts per million (or lower) level within the nitrogen, oxygen, carbon dioxide, water, and inert gas mixture, careful separation and identification is critical. VOC can derive from both exogenous sources (ex: pollutants) and endogenous biochemical processes. Here, we restrict our discussion of exhaled breath biomarkers to nitric oxide and VOC.

#### Nitric oxide

Nitric oxide (NO) is produced from the conversion of l-arginine to l-citrulline by a variety of lung resident cells as a result of the action of nitric oxide synthases (NOSs) (130). NO acts as a potent vasodilator but also inhibits vascular smooth muscle cell proliferation. Importantly, inhaled NO therapy is approved by the US Food and Drug Administration and European Medicine Evaluation Agency for the treatment of infants with persistent PH of the newborn who have acute hypoxemic respiratory failure (131). Clinically, the most extensively studied exhaled breath biomarker is fractional exhaled nitric oxide (FE_NO_) (132). Despite technical problems with standardization of its measurement, FE_NO_ is lower in patients with PH but rises in responders to therapy. Thus, serial FE_NO_ measurement may be useful. The measurement of NO may be complicated by genetic predisposition. In a study of Aymaran children (Aymara are an indigenous native nation who live in the Andes and Altiplano regions of South America), exhaled NO was actually lower (133). Thus, children who are protected from hypoxic PH due to high altitude do not apparently require increased respiratory NO synthesis. This may be due to NO biosynthesis by endothelium NOS3, by airway epithelium via NOS2, and/or by non-adrenergic non-cholinergic nerves via NOS1 (134). NO deficiency is a major therapeutic target in PH, primarily by stabilization of 3′,5′-cyclic guanosine monophosphate (cGMP) (phosphodiesterase type 5 inhibition) (135), and indirectly by prostacyclins and endothelin receptor antagonists (136). Indeed, a distinct class of emerging therapies for PH is based upon the antagonistic actions of asymmetric dimethylarginine (ADMA) regulating NO production (137). Elevated plasma ADMA has been shown to correlate to severity of idiopathic (138) and chronic thromboembolic PH in adults (139), and in children with congenital heart disease (140) and sickle cell disease with PH (141). Moreover, in one study of systemic heart failure associated with pulmonary venous hypertension, exhaled NO levels were in higher in hypertensives, but NO did not correlate to increased plasma ADMA (142). Therefore, plasma levels of the NO-ADMA axis may not be congruent with exhaled NO measures, making conclusions about biomarker utility somewhat murky. Despite the complexities of NO biology in the setting of PH, serial measurement of exhaled NO will likely continue to be a biomarker, particularly as a measure of response to a variety of therapies to treat PH.

##### Volatile organic compound

Recently, a mass spectrometry approach was used to identify several VOC that could distinguish PH patients from controls (143). In that study, exhaled ammonia was increased in PH patients and correlated with disease severity. Other studies have also demonstrated differences in endothelin-1, 6-keto PGF(1alpha), and NT-proBNP in exhaled breath condensates in individuals with PH compared to controls (144).

There has been a robust increase in the number of studies utilizing breath analysis in the past decade (“robust” as assessed by retrieval of 1301 articles in a January 2014 PubMed search for “exhaled biomarker,” 1042 published from 2003 to 2014, with only 258 publications involving children). Several aspects of exhaled breath analysis in the clinical setting make it particularly attractive to apply to the pediatric patient population. Breath tests are non-invasive and, in many cases, highly repeatable. Children are quite amenable to breathing treatments when fitted with fun masks and when clinicians make it fun. The same approaches could be potentially used to diagnose pediatric PAH and to monitor its progression and/or response to therapy.

### Urinary biomarkers

Besides peripheral blood, additional biological fluids are available that could offer prognostic value in pediatric PAH. Recently, Cracowski et al. prospectively studied serum biomarkers and urinary F_2_-isoprostane in 110 adults with PAH (145). Only urinary F_2_-isoprostane is independently associated with an increased 3-year hazard of death. Furthermore, urinary F_2_-isoprostane levels can be elevated in asymptomatic individuals predisposed to PAH, suggesting that their measurement may herald early stages of the disease. The source of F_2_-isoprostanes is unknown, as is their relevance to pulmonary vascular remodeling and increased PA pressure. Known to have pro-inflammatory properties and direct effects on both smooth muscle cells and ECs, they are generally considered markers of oxidative stress. Urinary F_2_-isoprostane levels could be measured in children at diagnosis of PH, and/or in children of families with histories of familial PAH who may be asymptomatic. Urinary cGMP levels were found to be higher in patients with PAH compared to asthma patients and controls (146). Urinary cGMP levels inversely correlated with cardiac index, likely a reflection of NO and BNP-related hemodynamic impairment.

## Specific Recommendations to Apply Adult Pulmonary Hypertension Biomarkers to Children

Biomarkers have the potential to be utilized as tools for identification of disease phenotype, patient response to therapy, and disease pathophysiology. Currently, the roles of several biomarkers have been identified in the pathophysiology of adult PAH, yet further characterization of these markers needs to be validated, clinical methods standardized, and engineering refined for development of robust assays. This level of investigation is not mirrored in pediatric PAH. A very limited number of studies have been conducted on elucidating biomarkers in children with PAH. It cannot be overstated that biomarkers in children with PAH are likely to be even more differentially influenced by additional factors compared with adults with PAH. Such factors include, but are not limited to: degree of physical activity, age in years and/or stage of development, gender differences, and nutritional status. Currently, several hurdles need to be overcome before we can advance to earlier diagnosis, more accurate prognostication, and improved therapy in children with PAH. With the exception of PAH associated with congenital abnormality, the diagnosis of PH in children is usually delayed, as it is in adults. Therefore, the most powerful biomarkers would be those that would identify individuals for whom the development of PAH is imminent or who are asymptomatic. Second, we need more sensitive and accurate biomarkers of disease progression that take into account developmental changes. Finally, we need biomarkers to identify which children will respond more favorably to one therapy or combinations of therapies and to confirm efficacy. For example, as imaging becomes faster, more powerful, and more precise, we anticipate that it will play an increasingly important role as a biomarker tool. The use of multiple biomarker panels assessed in large multi-center collaborations to increase cohort sizes will change the current paradigm of 100s of studies on a few kids to a few studies on 100s of kids. We recently summarized the current approach and diagnostic classification of PAH in children, as based on discussions and recommendations from the Pediatric Task Force of the fifth World Symposium on Pulmonary Hypertension (WSPH) in Nice, France (147). We outline our perspective on the use of biomarkers with regard to the classification, etiology, epidemiology and survival, diagnosis, treatment goals, and treatment of pediatric PH. In future discussions, we hope to reach agreement on the widespread adoption of specific sets of biomarkers discussed in this review, with a particular focus on those already identified as informative in adults with PH. We speculate that such a paradigm shift in experimental approach will translate into a transformative understanding of the natural history of PH in children.

## Author Contributions

Melanie J. Dufva, Ryan P. Delaney, Kelley L. Colvin wrote sections of the review. D. Dunbar Ivy and Kurt R. Stenmark and Kelley L. Colvin edited the review. Michael E. Yeager conceived of, wrote, and edited the review.

## Conflict of Interest Statement

The authors declare that the research was conducted in the absence of any commercial or financial relationships that could be construed as a potential conflict of interest.

## Abbreviations

CEC, circulating endothelial cell; CHD, congenital heart disease; CT, computed tomography; EC, endothelial cell; Echo, echocardiography; EPC, endothelial progenitor cell; MRI, magnetic resonance imaging; PAH, pulmonary arterial hypertension; PET, positron emission tomography; PH, pulmonary hypertension; PVR, pulmonary vascular resistance; RHC, right heart catheterization; RV, right ventricle; SPECT, single photon emission computed tomography; TDI, tissue Doppler imaging.
